# Global Variation in Hand Hygiene Practices Among Adolescents: The Role of Family and School-Level Factors

**DOI:** 10.3390/ijerph18094984

**Published:** 2021-05-07

**Authors:** Santosh Jatrana, Md. Mehedi Hasan, Abdullah A. Mamun, Yaqoot Fatima

**Affiliations:** 1 Centre for Rural and Remote Health, James Cook University, Mount Isa, QLD 4825, Australia; santosh.jatrana@jcu.edu.au; 2 School of Demography, The Australian National University, Canberra, ACT 2601, Australia; 3 Alfred Deakin Institute for Citizenship and Globalisation, Deakin University, Melbourne, VIC 3220, Australia; 4 Department of Public Health, University of Otago, Wellington 6021, New Zealand; 5 Institute for Social Science Research, The University of Queensland, Indooroopilly, QLD 4068, Australia; m.m.hasan@uq.net.au (M.M.H.); mamun@sph.uq.edu.au (A.A.M.); 6 The Australian Research Council Centre of Excellence for Children and Families over the Life Course (The Life Course Centre), The University of Queensland, Indooroopilly, QLD 4068, Australia

**Keywords:** hand hygiene, hand washing, global, adolescents, school children

## Abstract

While appropriate hand hygiene practices (HHP) are protective against infections, the paucity of evidence on global estimates and determinants of HHP in adolescents limits effective design and planning of intervention to improve HHP in young people. We examined the prevalence and correlates of HHP in adolescents. We used nationally representative data from the Global School-based Student Health Survey (2003–2017) from 92 countries. HHP were categorized as “appropriate”, “inappropriate” and “lacking” based on the information about “hand washing before eating”, “hand washing after using the toilet”, and “hand washing with soap”. Multinomial logistic regression analyses were used to assess the role of socio-demographic, health, lifestyle, school, and family-related variables in HHP. Among 354,422 adolescents (13–17 years), only 30.3% were found to practice appropriate hand hygiene. Multivariable models suggest that sedentary behavior (adjusted relative risk ratio (ARRR) 1.41, 95% CI 1.31–1.51)), and bullying victimization (ARRR 1.20, 95% CI 1.10–1.30) promoted inappropriate HHP. In contrast, parental supervision (ARRR 0.55, 95% CI 0.50–0.59) and parental bonding (ARRR 0.81, 95% CI 0.75–0.87) were protective against inappropriate HHP. From a policy perspective, hand hygiene promotion policies and programs should focus on both school (bullying, exercise) and family-level factors (parental supervision and parental bonding) factors.

## 1. Introduction

Over 150 years ago, Ignaz Semmelweiss first demonstrated the effectiveness of a seemingly simple intervention, hand washing, in preventing obstetrical nosocomial infections [1,2]. Since then, hand washing with soap and water at key times is heralded as one of the most cost-effective measures to reduce the global burden of gastrointestinal and respiratory diseases [3]. Post the emergence of COVID-19 there is a renewed emphasis on good hygiene practices, specifically hand washing with soap, for protecting against coronavirus disease (COVID-19) infection and breaking the chain of transmission [4,5]. The emphasis on washing hands with soap was mainly because much of the impact of hand hygiene practices on health is mediated through the use of soap in washing hands. Hand washing with soap was listed as one of three key behaviors in the global Water Supply and Sanitation Assessment 2000 report by WHO that are of greatest likely benefit to health, particularly in developing countries [6]. Moreover, a systematic review reported good evidence that hand washing with soap has a similar impact on diarrhea in both industrialized and developing countries, where water supply and sanitation differ greatly [3]. This study provided good evidence to justify the promotion of hand washing with soap.

Never before have hand hygiene practices ever received so much global attention as in the current time. In the past few months, a significant amount of financial and human resources have been invested in various campaigns and initiatives to improve the awareness and practice of appropriate hand hygiene [7,8]. It is important to understand that the success of these initiatives, which are primarily aiming for behavior change, will depend on a thorough mapping of current hand hygiene behaviors and information on attitudes, barriers, and enablers for hand hygiene. This information will help to identify the gaps in current strategies and opportunities for improvements to develop and deliver effective interventions.

While examining the population hand hygiene practices is of interest in its own right, it is particularly important to explore these practices in young people, as they are the “silent carriers” who unknowingly play a major role in community transmission of infections [9]. It is well established that adolescence, a sensitive phase of life [10], exerts significant influence in shaping long term health behaviors [11]. Extant research indicates that hand hygiene is suboptimal in young people [12,13]. Therefore, instilling appropriate hand hygiene in young people will help them in continuing these practices in adult life. Moreover, school-based intervention strategies—including school closures and hygiene and health intervention programs have been shown to be cost-effective in reducing the spread of infectious diseases [14]. Hence, it is important to assess the prevalence and the determinants of hand washing behavior among adolescents attending schools globally.

Unfortunately, despite the established cost-effectiveness of hand hygiene in preventing and controlling the spread of infections, there is a paucity of evidence on global estimates, variations and determinants of hand hygiene practices among adolescents attending schools globally. The limited research on hand hygiene practice has many limitations. First, only a few studies have produced evidence from nationally representative studies [15,16]. Brauer and colleagues made an important contribution to the literature on hand washing by estimating hand washing station with water and soap access to inform use of hand washing in the prevention of COVID-19 transmission [17]. However, access to hand washing facilities does not denote actual hand washing practices [18,19]. Moreover, their “estimates did not include access to hand washing facilities in non-household settings such as schools, workplaces, health care facilities, and other public locations such as markets” [17].

Secondly, the bulk of research in this area has been carried out mainly on individual countries or a group of countries [20,21,22], and there is a paucity of evidence on global estimates and variations. A global-level assessment of hand hygiene practices is essential to assess the nature and extent of cross-country and regional level variations in hand hygiene practices across the world [18]. That, in turn, will inform the tailoring and ramping up the hand hygiene promotional activities in countries with subpar performance and help in maximizing the impact of hand hygiene promotional activities.

Third, past evidence on hand hygiene practices in adolescents is limited to hand washing practices at key times and hand washing with soap separately [11,15,17,19,20,21,23,24]. In the context of COVID-19 and beyond, merely washing hands will not be enough to minimize the risk of infection transmission in the community. The key indicator for hand hygiene assessment should combine both “when” (i.e., at key times) and “how” (i.e., with soap) for hand washing. Only then we can identify the level and intensity of interventional that would be required for different groups.

Addressing these gaps in the literature, this paper sets out to utilize nationally representative data to examine global hand washing practices in adolescents, assess regional and country-level variations in adolescents’ hand hygiene practices, and identify the key hand washing motives and barriers to hand washing practices that are context-specific such as socio-demographic, lifestyle, and family-related variables in driving adolescents’ hand hygiene practices.

Our work is driven by behavioural change theories that have been applied to infection-control practices [25]. There exist multiple theories that have most strongly influenced behavioural prevention research, such as the theory of reasoned action [26], and the theory of planned behavior [27]. The underlying premise of these theories is based upon the understanding of the factors influencing ‘a person’s decision to perform (or not perform) a given behavior’ [27] (for example, appropriate hand washing in our case). These theories provide a structure to select our independent variables, define our analytical approach, and serve as the guide to discuss our findings. In accordance with the constructs of these theories and with previous research [21,28,29,30], we consider both school-level factors and family-level factors in our analysis. The school-level factors such as exercise and bullying can play an important role in hand hygiene behavior [21,31]. Indeed, previous research has shown significant oral and hand washing behavior changes among children after a school-based educational intervention [31,32,33,34]. School-based behavioral interventions can support children to develop independent and healthy hygiene behaviors, which can endure throughout adulthood. Moreover, children are likely to bring behavioral changes by passing on and communicating the health message received at school to their parents and family members, including their siblings [23,24,31]. The family-level factors, such as parental supervision and parental bonding, play an important part in the development of their adolescent children via shared time and shared information [29]. Individual socioeconomic factors have shown a significant association with hand washing behavior among school children [28].

## 2. Materials and Methods

### 2.1. Data

This study used data from the Global School-based Student Health Survey (GSHS) that are conducted in low to middle- and high-income countries. In collaboration with a number of organizations, the GSHS was jointly developed by the World Health Organization and the United States Centre for Disease Control and Prevention (CDC). The GSHS employed a two-stage cluster random sampling strategy to collect information on ten core questionnaire modules that addressed the leading causes of morbidity and mortality in adolescents. In brief, the modules included tobacco use, alcohol use, drug use, dietary behaviors, hygiene, physical activity, sexual behaviors, unintentional injury and violence, and mental health. The GSHS used uniform methodology and questionnaire to allow cross-country comparison of indicators. Project-related design, organization, and implementation have been described elsewhere [35].

### 2.2. Study Population

We downloaded the GSHS data of 100 countries and used the most recent data of those countries that collected data in multiple rounds. Among all the countries, 92 countries had available data (survey conducted during 2003–2017) on hand washing practices. The final sample included survey data from 354,422 adolescents (13–17 years) from 92 countries.

### 2.3. Measurements

#### 2.3.1. Outcome Variables

The data on the following three hand hygiene variables were collected. The response to each question was recorded as “never”, “rarely”, “sometimes”, “most of the time”, and “always”. In this study, we recoded these responses variable into three categories, i.e., “rarely/never”, “sometimes/mostly”, and “always”.

Hand washing before eating: The GSHS asked respondents the question “During the past 30 days, how often did you wash your hands before eating?”

Hand washing after using the toilet: The GSHS asked respondents the question “During the past 30 days, how often did you wash your hands after using the toilet or latrine?”

Hand washing using soap: The GSHS asked respondents the question “During the past 30 days, how often did you use soap when washing your hands?”

Hand hygiene practice: As mentioned in the introduction, in the context of COVID-19 and beyond, merely washing hands will not be enough to minimize the risk of infection transmission in the community. The key criteria for hand hygiene assessment should be hand washing with soap for the recommended duration (≥20 s) [36]. To overcome the limitations of the bulk of the studies on this topic, where hand washing at key times and hand washing with soaps are separately explored, we created a composite indicator to assess hand hygiene practices by combing both “when” and how” for hand washing. However, the key criteria to define appropriate hand hygiene practices was the use of soap to wash hands [36]. Therefore, those who were always using soap to wash hands were categorized as following appropriate hand hygiene, whereas those who did not always use soap for hand washing were categorized as practicing inappropriate hand hygiene, and those who never used soap were categorized as lacking hand hygiene practices. The rationale behind creating the three groups was to assess hand hygiene practices in adolescents and identify the level and intensity of interventional that would be required for different groups. Study participants were grouped into the following three categories:

Appropriate hand hygiene practices: The adolescents were deemed as practicing appropriate hand hygiene if they selected “always” for three variables mentioned above, i.e., they reported “always” using the soap to wash their hands, “always” before eating the food and “always” after using the toilet.

Inappropriate hand hygiene practices: The adolescents were deemed as practicing inappropriate hand hygiene if they selected “sometimes/mostly” for washing hands with soap and selected any of the response options, i.e., “rarely/never”, “sometimes/mostly”, and “always” for the remaining two variables (i.e., before eating the food and after using the toilet).

Lack of hand hygiene practices: The adolescents were deemed as lacking hand hygiene if they selected “never/rarely” for washing hands with soap and selected any of the response options, i.e., “rarely/never”, “sometimes/mostly” and “always” for the remaining two variables.

#### 2.3.2. Independent Variables

The selection of independent variables is informed by the literature and guided by the theories of health behavior change, particularly the theories of behavioral changes for hygiene promotion [21,22,37,38,39]. The independent variables were chosen in such a way that they could capture a wide range of demographic, economic, family, school, mental and behavioral factors affecting hand hygiene practice, such as age, sex, socioeconomic status (SES), physical activity, sedentary behavior, smoking, alcohol consumption, bullying victimization, loneliness, parental supervision, and parental bonding. The definition of each exposure variable is presented below.

Age: The GSHS question on age was “How old are you?”. The responses for this question were “11 years old or younger”, “12 years old”, “13 years old”, “14 years old”, “15 years old”, “16 years old”, “17 years old”, and “18 years old or older”. Though GSHS generally collected data from respondent 13–17 years of age, some countries included responses from adolescents out of this age range. However, we considered adolescents 13–17 years of age only to study the participants drawn from the same age cohort.

Socioeconomic status (SES): The GSHS question for the proxy of socioeconomic status was, “During the past 30 days, how often did you go hungry because there was not enough food in your home?” The responses for this question were “Never”, “Rarely”, “Sometimes”, “Most of the time” and “Always”. Aligning with the previous GSHS studies [40], a respondent was considered to belong to the “Average” SES group if his or her responses were “Never” or “Rarely”. If the responses were from “Sometimes” to “Always”, then the respondents were recorded to belong to the “Below average” SES group.

Physically active: The GSHS question for physically active was, “During the past 7 days, on how many days were you physically active for a total of at least 60 min per day?”. The responses for this question were “0 days”, “1 day”, “2 days”, “3 days”, “4 days”, “5 days”, “6 days”, and “7 days”. If the respondents were physically active for at least 5 days during the past 7 days, then he or she was considered physically active. Otherwise, the respondents were treated as not physically active [22].

Sedentary behavior: The GSHS question on sedentary behavior was, “How much time do you spend during a typical or usual day sitting and watching television, playing computer games, talking with friends, or doing other sitting activities?” The responses for this question were recorded as <1, 1–2, 3–4, 5–6, 7–8, and ≥8 h/day. Adolescent’s time spending at school and on doing homework was excluded from this time count. Considering the health risks, we created a dichotomous variable and considered adolescents to engage in a sedentary activity if s/he spends ≥3 h/day, otherwise not [22].

Smoking: The GSHS asked respondents the question, “During the past 30 days, on how many days did you smoke a cigarette?” If a participant smoke cigarette for at least 1 day during the past 30 days, s/he is considered smoking cigarettes [41].

Alcohol consumption: The GSHS asked respondents the question, “During the past 30 days, on how many days did you have at least one drink containing alcohol?” If a participant drank for at least 1 day during the past 30 days, s/he is considered to consume alcohol [41].

Bullying victimization: To measure bullying, the GSHS asked respondents the following question “During the past 30 days, on how many days you were bullied?” Before asking this question, the respondents were given a statement first to read the definition of bullying. After that, they were asked how many days they experienced bullying in the last 30 days. The responses were recorded as “0 days”, “1 or 2 days”, “3 to 5 days”, “6 to 9 days”, “10 to 19 days”, “20 to 29 days”, and “All 30 days”. Aligning with previous research [40,42], we created a dichotomous variable to define bullying victimization, and a respondent is defined as being bullied if s/he reported experiencing bullying at least once in the past 30 days preceding the survey. We coded the dichotomous responses as 0 “No”, meaning 0 days of experiencing bullying and 1 “Yes”, meaning the experience of bullying for at least once.

Loneliness: The GSHS question was, “During the past 12 months, how often have you felt lonely?”. The responses were “Never”, “Rarely”, “Sometimes”, “Most of the time”, and “Always”. If a respondent “Most of the time” or “Always” felt lonely, we considered him or her to feel lonely. Otherwise, he or she was not considered as to feel lonely [41].

Parental supervision: The GSHS asked the following question “During the past 30 days, how often did your parents or guardians check to see if your homework was done?”. The responses were “Never”, “Rarely”, “Sometimes”, “Most of the time”, and “Always”. We considered adolescents under parental supervision if their response to this question was either “Most of the time” or “Always”, otherwise he or she was considered not to have parental supervision [22,41].

Parental bonding: The GSHS asked the following question “During the past 30 days, how often did your parents or guardians really know what you were doing with your free time?”. The responses for this question were “Never”, “Rarely”, “Sometimes”, “Most of the time”, and “Always”. We used this variable to define parental bonding. We considered adolescents to have bonded with their parents if their response to this question was either “Most of the time” or “Always”, and adolescents was considered not to have bonded with parents if their response was either “Never” or “Rarely” or “Sometimes” [22,41].

### 2.4. Statistical Analyses

The prevalence of hand hygiene practices and background characteristics of the study participants were explored by using univariate analysis. We applied bivariate analysis to examine the differentials in the prevalence of hand hygiene practices across the background characteristics of adolescents. Statistical significance in the bivariate analysis was detected by applying the Chi-square test. As our dependent variable (DV) had three categories, we applied multiple multinomial logistic regression analyses to examine the association of background characteristics with hand hygiene practices. In conducting multiple regression analysis, we fitted a simple regression model to assess whether the independent variables (IV) are associated with the DV. We included all the IVs in the multiple regression model, given their evidence of having a significant association with DV in simple regression models. Before entering the IV in the multiple regression model, we checked the multicollinearity among the IVs and found the absence of multicollinearity (variance inflation factor <2). The results of the regression analysis were presented in terms of relative risk ratio (RRR) with the respective 95% confidence interval (CI). As surveys were conducted at different time points (survey years) across different countries, we considered the time point as an independent variable in regression models to adjust the effect of time variations. Due to the hierarchical structure of the GSHS data, we considered the cluster sampling design and sampling weight of the GSHS through defining “*svyset*” in Stata and used “*svy*” as a prefix in all analyses. The statistical significance was defined at a 5% level (*p*-value < 0.05). Stata (version 15) was used for data analysis.

## 3. Results

### 3.1. Sample Characteristics

Among 354,422 adolescents analyzed globally, nearly half of the adolescents (48.6%) were female. Almost one-third of the adolescents (31.7%) belonged to below averaged SES, and a little less than one-third of the adolescents (31.8%) were physically active. A significant number of the adolescents (34.9%) were bullied by their peers, and one out of ten (11.4%) felt lonely. Nearly two of every five adolescents reported receiving parental supervision (39%) and bonding with parents (41.5%). There were variations in sample characteristics across regions. See Table 1 for details.

### 3.2. Hand Hygiene Practices

Globally, only 30.3% (95% CI: 29.2–31.4%) reported practicing appropriate hand hygiene. Whereas 60.4% (95% CI: 59.3–61.5%) and 9.4% (95% CI: 8.8–9.9%) of the adolescents reported practicing inappropriate hand hygiene and lack of hand hygiene, respectively. Item-specific pooled estimates highlighted that out of the total sample, only 56.7% (95% CI: 55.2–58.1%) reported “always” washing their hands before eating, and 68.3% adolescents (95% CI: 67.3–69.4%) reported “always” washing their hands after using the toilet, while only 45.3% (95% CI: 44.1–46.5%) reported “always” using soap to wash their hands.

**Figure 1:**
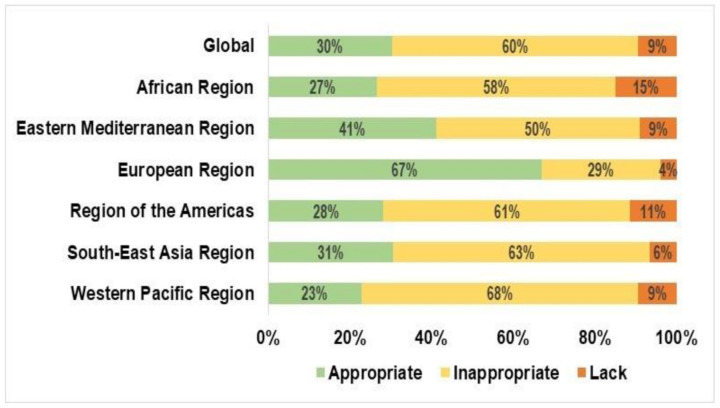
Regional variation in adolescents’ hand hygiene practices across the globe, based on the Global School-based Student Health Survey data (2003–2017).

The prevalence of hand hygiene practices varied across the countries and regions. At the regional level, inappropriate hand hygiene practices were reported to be highest in adolescents from the Western Pacific region (67.9%, 95% CI: 65.6–70.1%) and lowest in adolescents from the European region (29.1%, 95% CI: 25.9–32.4%) (Figure 1).

At country level, the prevalence of inappropriate hand hygiene practices in adolescents was reported to be highest in Vietnam (78.7%, 95% CI: 76.8–80.5%) followed by Solomon Islands (78.7%, 95% CI: 74.9–82.0%) and Cambodia (78.4%, 95% CI: 76.0–80.6%), and lowest in Honduras (28.5%, 95% CI: 26.2–30.9%), followed by Macedonia (29.1%, 95% CI: 25.9–32.4%) and Senegal (34.7%, 95% CI: 30.6–39.0%) (Supplementary Figure S1). In addition, lack of hand hygiene practices were highest in adolescents from Honduras (58.7%, 95% CI: 55.3–62.1%), Benin (23%, 95% CI: 18.8–27.7%), and Sudan (22.8%, 95% CI: 19.1–27.0%), and lowest in adolescents from Lebanon (2.3%, 95% CI: 1.9–2.8%), Cambodia (2.7%, 95% CI: 2.2–3.4%), and Mongolia (3.1%, 95% CI: 2.5–3.8%).

### 3.3. Correlate of Hand Hygiene Practices

Hand hygiene practices varied significantly (*p*-value < 0.05) across adolescent’s age, sex, SES, physical activity, sedentary behavior, smoking status, alcohol consumption, bullying victimization, loneliness, parental supervision, and parental bonding (Table 2). The results from pooled multivariable multinomial logistic regression model for global data (Table 3) showed that sedentary behavior was associated with an increased odds of inappropriate hand hygiene practices (adjusted RRR (ARRR) 1.41, 95% CI 1.31–1.51, *p*-value < 0.001). Adolescents who were bullied at school had higher odds of inappropriate hand hygiene practices than their counterparts (ARRR 1.20, 95% CI 1.10–1.30, *p*-value < 0.001). Parental supervision (ARRR 0.55, 95% CI 0.50–0.59, *p*-value < 0.001) and bonding with parents (ARRR 0.81, 95% CI 0.75–0.87, *p*-value < 0.001) were associated with lower odds of inappropriate hand hygiene practices. 

## 4. Discussion

### 4.1. Main Findings and Contributions

In this study, we examined the prevalence and correlates of hand hygiene practices in adolescents across the globe. There are three major findings: first, irrespective of socioeconomic disparities, adolescents’ hand hygiene practices in most of the 87 countries are suboptimal. Second, there is a substantial cross-national variation in the practice of appropriate hand hygiene in adolescents. Third, bullying victimization is associated with a higher risk of inappropriate hand hygiene, while physical activity, parental supervision, and bonding are associated with a lower risk of inappropriate hand hygiene practices. Since sedentary behaviors are strongly associated with lacking HHP, efforts are needed to promote HHP in physically inactive adolescents. Furthermore, as sedentary behaviors and HHP are strongly associated with each other, interventions targeting sedentary behavior may subsequently improve HHP in adolescents; therefore, school level physical activity promoting interventions can provide opportunities to improve HHP in students.

These findings highlight the need to further improve the effectiveness of current hand hygiene promoting programs with consideration for both schools (bullying, exercise) and family level (parental supervision and parental bonding) influencers on hand washing behaviors. Therefore, while devising policies and programs targeted at improving physical activity and controlling bullying is crucial to enhance hand washing practices among adolescents, parent–child bonding and shared time are crucial in promoting children’s hand hygiene as well. These results imply that public health policies need to be targeted at not only providing health education but at increasing parent–child bonding and shared time in order to promote children’s health more effectively.

### 4.2. Interpretation and Comparability

While access to hand washing facilities and knowledge of proper hygiene is important for practicing hand washing [43], the knowledge–behavior gap [44] is a major reason for sub-optimal hand hygiene practices. For example, Rabbi and Day found that a majority (90%) of respondents had knowledge about hand washing with soap before eating and after defecation, but only 21% and 88% of respondents reported doing so, respectively [44]. Systematic integration of health and hygiene education in school curriculum and peer-led behavior change initiatives could be an appropriate strategy to reduce inappropriate hand hygiene practices. Clearly, 30.3% of adolescents who are following appropriate hand hygiene need the lowest intensity of intervention to ensure the continuation of current hand hygiene practices. Whereas 60.5% of adolescents who are reporting “sometimes/mostly” for washing hands with soap appear to have access to soap (and presumptively water), and despite that, they are not “always” using soap to wash their hands. Therefore, for this group, multi-focused intervention, particularly targeting attitude and behavior, is needed. The remaining 9.4% of the adolescents reported lack of hand hygiene, their reporting of “rarely/never” washing their hand with soap could be due to the lack of access to soap or water in the households or school [45], lack of clean toilets in school [12], inconveniently located sinks [46], inadequate knowledge [12], or poor attitude and behavior for practicing hand hygiene [47]. Previous research has also highlighted that in addition to awareness and self-efficacy, the availability of soap and clean water can significantly improve hand hygiene practice in young people [28,48]. Therefore, the group reporting the lack of hand hygiene will require multilevel and multi-focused intervention to ensure access to facilities, adequate awareness, and the right behavior to achieve appropriate hand hygiene practices.

Our results further highlight a huge cross-national, as well as regional variation in the prevalence of appropriate hand hygiene practices. For example, the prevalence of appropriate hand hygiene practices in adolescents was highest in the European region (66.9%) and lowest in the Western Pacific region (22.8%). Similarly, the prevalence of appropriate hand hygiene varied across countries ranging from 66.9% in Macedonia (European region) to 7% in Vanuatu (Western Pacific region). Such variation may reflect “the relative economic development of the countries, variations in international aid, the different emphasis of public health programs and cultural variations” [30]. The large cross-national differences do point to the need for more comprehensive hand hygiene promotional activities in countries where a high proportion of students reported inappropriate hand hygiene practices.

The multivariable analysis showed that both school (exercise, bullying) and family-level factors (parental supervision and parental bonding) influence hand hygiene practices. For example, adolescents with a sedentary lifestyle were more likely to have inappropriate and lack hand hygiene practice. These results are in accordance with another study based on the GSHS data, which found sedentary behavior to be associated with sub-optimal hand hygiene behavior [21]. We also found that adolescents who were bullied were more likely to have inappropriate and lack hand hygiene practice than those who were not bullied. The fear of being bullied may discourage children from using toilets and drinking and hand washing facilities [38]. According to a World Bank report from Kenya, hand washing facilities in most schools are too high to reach [49]. Younger children find it even harder than usual to reach the hand washing facilities and may even be bullied and pushed by older children when they line up to wash their hands [49]. As adolescents spend a major proportion of the day at school, ensuring supervision of the hand wash facilities may help in improving hand hygiene practice and reduce the other negative impacts of bullying as well.

The role of parental supervision in hand hygiene practices is also established in other studies where the lack of parental supervision and parental bond was shown to increase the risk of sub-optimal hand washing with soap [21]. A large body of evidence indicates that parents play an important part in the development of their adolescent children [50]. Increased parental supervision and bonding may be proxies of time spent by parents with their adolescent children. It is possible that as parents spend more time with their children, they may have more chances to talk with their children about their health-related concerns or belief [29].

It is noteworthy that there is no significant gender difference in hand washing behavior. Our results are in line with studies, which found gender not associated with hand washing behavior [20,28]. Several studies, however, have shown that girls have better hand hygiene habits than boys [30]. These contradictory findings do point to the fact that gender differences in hand hygiene may not be universal, and, hence, there is a need to introduce hand hygiene initiatives in adolescents irrespective of gender.

### 4.3. Strength and Limitations

A major strength of the study is that the large population-wide samples drawn from randomly selected schools with a similar target population, sampling frame, standardized methods, and survey questions allowing comparison of hand hygiene behavior in adolescents across the globe. Compared with previous studies that only focused on hand washing, we used a more robust measure to assess hand hygiene. However, we acknowledge that in addition to “how” and “when” we also needed to include the information on “how long” for adequately assessing hand hygiene practices. Notably, in the context of the current COVID-19 situation where hand washing with soap is recommended ≥20 s, the lack of data on the duration of hand washing is a limitation of our study. Considering that a significant number of adolescents are reported to have low awareness of appropriate duration for hand washing [12], we anticipate that lack of information on hand washing duration might have led to an overestimation of appropriate hand hygiene practices in our study. Additionally, since the hand hygiene practices were measured by self-reported data, our estimates are prone to reporting error and recall bias. The GSHS only includes school-going adolescents who may not be representative of all adolescents in a country. Hence, as with all school-based research, the findings may not apply to adolescents who do not attend school. We presumed that those adolescents who reported “mostly/sometimes” hand washing with soap, as opposed to “always” had access to soap and water, but specific questions on the access to soap and water, hand hygiene knowledge and aptitude are needed for reliable assessment of hand hygiene practices and drivers. As the GSHS administered in 2 European countries (i.e., Tajikistan and Macedonia), we have information on HHP for Macedonia only. Therefore, we acknowledge that this single country analysis may not be able to represent a region. Other countries that have available information on HHP must need to be analyzed to better understand the HHP scenario in this region.

## 5. Conclusions

Our study demonstrated suboptimal hand hygiene practice in adolescents and showed that both school (bullying, exercise) and family-level factors (parental supervision and parental bonding) influence hand hygiene practices. Therefore, the design and development of hand hygiene promotion policies and programs should ensure bullying prevention at schools, physical activity promotion in schools and parental involvement in hand hygiene activities. Our results imply that public health policies need to be targeted at not only providing health education but at increasing parent–child bonding and shared time in order to promote children’s health more effectively.

## Figures and Tables

**Table 1:** Sample characteristics.

	Global	African Region	Eastern Mediterranean Region	European Region	Region of the Americas	South-East Asia Region	Western Pacific Region
**Age**							
13 years	65,546 (22.4)	7767 (20.2)	15,160 (26.9)	455 (26.8)	18,294 (21.7)	9876 (27.4)	13,994 (13.2)
14 years	83,515 (28.5)	10,969 (24.9)	18,728 (29.9)	448 (26.8)	24,125 (29.5)	12,203 (31.6)	17,042 (24.9)
15 years	78,710 (24.5)	12,629 (27.1)	16,730 (25.1)	569 (25.1)	21,382 (27.9)	10,599 (21.9)	16,801 (25)
16+ years	79,999 (24.6)	15,822 (27.8)	13,697 (18.1)	539 (21.3)	15,526 (20.9)	11,580 (19.1)	22,835 (36.9)
**Sex**							
Male	168,504 (51.4)	27,753 (51.6)	36,500 (53.6)	1033 (52.3)	41,780 (49.7)	24,040 (52.1)	37,398 (48.8)
Female	181,673 (48.6)	29,010 (48.4)	37,379 (46.4)	1053 (47.7)	45,963 (50.3)	27,402 (47.9)	40,866 (51.2)
**SES**							
Average	240,305 (68.3)	28,225 (61.9)	55,412 (75.5)	1986 (94.7)	71,995 (83.2)	29,840 (61.9)	52,847 (70.1)
Below average	94,731 (31.7)	20,308 (38.1)	18,528 (24.5)	108 (5.3)	16,063 (16.8)	14,202 (38.1)	25,522 (29.9)
**Physically active**							
No	215,791 (68.2)	26,979 (65.8)	45,045 (70.7)	503 (23.6)	57,038 (71.6)	31,921 (66.9)	54,305 (69.3)
Yes	110,649 (31.8)	18,378 (34.2)	18,728 (29.3)	1459 (76.4)	29,526 (28.4)	19,160 (33.1)	23,398 (30.7)
**Sedentary behavior**							
<3 h	219,251 (70.6)	34,015 (73.7)	46,619 (74.1)	922 (47.8)	49,301 (64)	37,870 (73.2)	50,524 (63.9)
≥3 h	120,231 (29.4)	15,822 (26.3)	25,915 (25.9)	1092 (52.2)	36,877 (36)	13,218 (26.8)	27,307 (36.1)
**Smoking**							
No	279,620 (91)	43,244 (92.5)	64,261 (92.1)	1700 (85.8)	58,259 (83.8)	43,099 (90.8)	69,057 (91.1)
Yes	37,220 (9)	4014 (7.5)	7942 (7.9)	324 (14.2)	11,256 (16.2)	5187 (9.2)	8497 (8.9)
**Alcohol consumption**							
No	196,182 (85.6)	34,675 (86.3)	7542 (90.7)	1088 (60.6)	48,977 (67.2)	38,075 (93.7)	65,825 (79.7)
Yes	57,827 (14.4)	8028 (13.7)	1007 (9.3)	794 (39.4)	32,651 (32.8)	4608 (6.3)	10,739 (20.3)
**Bullying victimization**							
No	212,727 (65.1)	26,507 (55.3)	39,852 (54.5)	1749 (90)	62,339 (70.9)	28,402 (72.1)	53,878 (68.1)
Yes	102,063 (34.9)	22,349 (44.7)	24,825 (45.5)	193 (10)	21,536 (29.1)	12,695 (27.9)	20,465 (31.8)
**Loneliness**							
No	296,365 (88.6)	41,885 (86.9)	57,896 (84.2)	1954 (93.6)	77,867 (89.7)	46,657 (92)	70,106 (87.8)
Yes	40,834 (11.4)	7240 (13.1)	11,071 (15.8)	144 (6.4)	9763 (10.3)	4728 (8)	7888 (12.2)
**Parental supervision**							
No	171,657 (61)	17,140 (51.9)	34,364 (55.7)	No obs	41,997 (59.1)	30,110 (57.8)	48,046 (75.3)
Yes	104,788 (39)	12,751 (48.1)	29,069 (44.3)	No obs	25,146 (40.9)	20,680 (42.2)	17,142 (24.7)
**Parental bonding**							
No	152,668 (58.5)	18,686 (62.5)	34,723 (57.6)	No obs	32,536 (50.8)	27,440 (55)	39,283 (65)
Yes	122,459 (41.5)	11,020 (37.5)	28,458 (42.4)	No obs	34,159 (49.2)	23,045 (45)	25,777 (35)

Results in the cells are presented in terms of sample size followed by weighted prevalence in the parenthesis, *n* (%). No obs stands for “No observation”.

**Table 2:** Factors associated with adolescents’ hand hygiene practices across the globe, based on the Global School-based Student Health Survey data (2003–2017).

Variable	Hand Hygiene Practices			
	Lacking	Inappropriate	Appropriate	*p*-Value
**Age**				<0.0001
13 years	8.4%	58.1%	33.5%	
14 years	8.6%	59.4%	32%	
15 years	9.5%	59.8%	30.6%	
16+ years	10.7%	64.2%	25.1%	
**Sex**				<0.0001
Male	10.6%	60.6%	28.8%	
Female	7.9%	60.2%	31.9%	
**SES**				<0.0001
Average	9.2%	59.2%	31.6%	
Below average	9.4%	63.1%	27.5%	
**Physically active**				<0.0001
No	9.2%	62.4%	28.6	
Yes	9.9%	58.9%	31.2%	
**Sedentary behavior**				<0.0001
No	8.7%	58.6%	32.7%	
Yes	10.6%	65.1%	24.3%	
**Smoking**				<0.0001
No	8.6%	60.4%	31.1%	
Yes	15.6%	62.0%	22.4%	
**Alcohol consumption**				<0.0001
No	8.3%	62.6%	29.1%	
Yes	13.8%	67.2%	19.0%	
**Bullying victimization**				<0.0001
No	7.5%	60.3%	32.3%	
Yes	11.3%	62.5%	26.3%	
**Loneliness**				<0.0001
No	9.0%	61.1%	29.9%	
Yes	11.2%	61.2%	27.6%	
**Parental supervision**				<0.0001
No	11.1%	65.8%	23.1%	
Yes	6.2%	53.9%	39.9%	
**Parental bonding**				<0.0001
No	11.2%	63.4%	25.4%	
Yes	6.4%	58.0%	35.6%	

**Table 3:** Time and cluster adjusted correlates of adolescents’ hand hygiene practices across the globe, based on the Global School-based Student Health Survey data (2003–2017).

	Inappropriate vs. Appropriate (Ref)		Lacking vs. Appropriate (Ref)	
	RRR (95% CI)	ARRR (95% CI)	RRR (95% CI)	ARRR (95% CI)
**Age**				
13 years				
14 years	1.01 (0.94–1.08)	0.93 (0.84–1.03)	1.00 (0.89–1.14)	0.87 (0.72–1.06)
15 years	1.05 (0.96–1.14)	0.91 (0.79–1.04)	1.14 (0.97–1.34)	1.03 (0.80–1.33)
16+ years	1.19 (1.10–1.29) ***	1.00 (0.89–1.13)	1.46 (1.26–1.68) ***	1.20 (0.96–1.5)
**Sex**				
Male				
Female	0.86 (0.82–0.9) ***	0.93 (0.86–1.01)	0.66 (0.60–0.73) ***	0.80 (0.70–0.92) **
**SES**				
Average				
Below average	1.22 (1.15–1.29) ***	0.98 (0.90–1.07)	1.15 (1.05–1.27) **	0.70 (0.59–0.82) ***
**Physically active**				
No				
Yes	0.9 (0.86–0.95) ***	0.89 (0.83–0.96) **	1.00 (0.91–1.11)	0.99 (0.84–1.16)
**Sedentary behavior**				
No				
Yes	1.40 (1.33–1.46) ***	1.41 (1.31–1.51) ***	1.61 (1.49–1.74) ***	1.57 (1.37–1.80) ***
**Smoking**				
No				
Yes	1.44 (1.31–1.59) ***	1.06 (0.9–1.23)	2.67 (2.17–3.29) ***	1.60 (1.09–2.36) *
**Alcohol** **consumption**				
No				
Yes	1.49 (1.39–1.60) ***	1.18 (1.07–1.30) **	2.39 (2.05–2.79) ***	1.31 (1.06–1.61)
**Being bullied**				
No				
Yes	1.40 (1.33–1.48) ***	1.20 (1.10–1.30) ***	2.07 (1.86–2.31) ***	1.69 (1.44–2.0) ***
**Loneliness**				
No				
Yes	1.11 (1.05–1.18) ***	1.03 (0.92–1.15)	1.34 (1.21–1.48) ***	1.06 (0.86–1.30)
**Parental supervision**				
No				
Yes	0.51 (0.48–0.54) ***	0.55 (0.50–0.59) ***	0.32 (0.29–0.36) ***	0.46 (0.39–0.54) ***
**Parental bonding**				
No				
Yes	0.67 (0.64–0.71) ***	0.81 (0.75–0.87) ***	0.40 (0.36–0.44) ***	0.55 (0.48–0.63) ***

RRR = Relative risk ration, ARRR = Adjusted RRR, CI = Confidence Interval, *** if *p* < 0.001, ** if *p* < 0.01, * if *p* < 0.05.

## Data Availability

All the estimates are presented in the manuscript. Raw data are publicly available and can be accessed through https://www.who.int/ncds/surveillance/gshs/datasets/en/.

## Supplementary Materials

The following are available online at https://www.mdpi.com/article/10.3390/ijerph18094984/s1, Figure S1: Distribution of sample across study regions and countries.
