# Supplementary figures and images for: Global Variation in Hand Hygiene Practices Among Adolescents: The Role of Family and School-Level Factors
Source: Int J Environ Res Public Health. 2021 May 7;18(9):4984. doi: 10.3390/ijerph18094984 (PMC8125682; doi:10.3390/ijerph18094984)

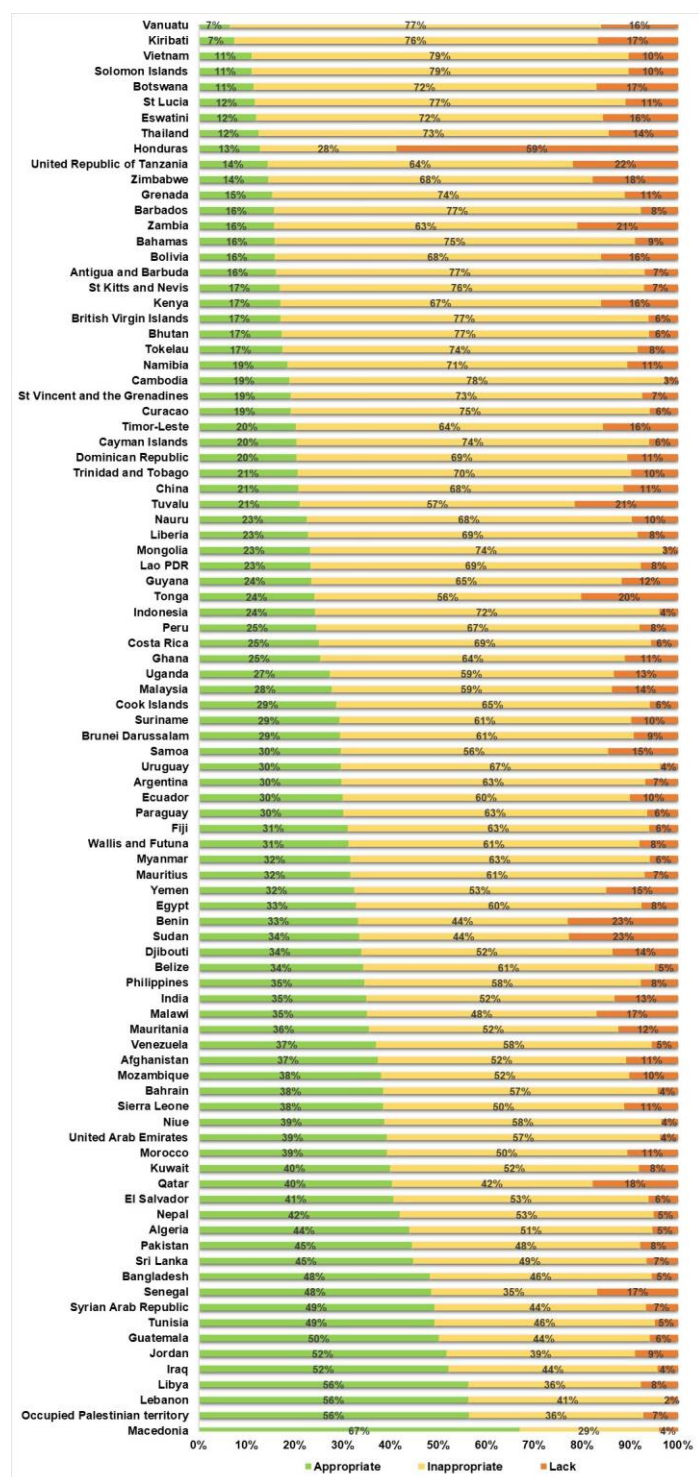

Figure S1.

Supplement: Supplementary file 1 [file ijerph-18-04984-s001.zip › ijerph-1208603-suppementary.pdf]
